# Salvaging Early EndoButton Migration in PCL Reconstruction: A Case Report Describing a Novel Technical Solution for Femoral Tunnel Complications

**DOI:** 10.1155/cro/3787522

**Published:** 2026-08-23

**Authors:** Daichi Ishimaru, Kazuki Sohmiya, Nobuo Terabayashi, Kazu Matsumoto

**Affiliations:** ^1^ Department of Orthopaedic Surgery, Gifu Seiryu Hospital, Gifu, Japan

**Keywords:** cortical button, posterior cruciate ligament, posterior cruciate ligament reconstruction, suspensory fixation

## Abstract

**Introduction:**

Recently, the number of posterior cruciate ligament (PCL) reconstruction surgeries has been increasing. A rare complication of early intratunnel migration of a suspensory cortical button into the femoral tunnel after PCL reconstruction was observed.

**Case Presentation:**

A 51‐year‐old man presented with an isolated PCL rupture and underwent PCL reconstruction surgery. A quadrupled semitendinosus autograft was used; femoral fixation was achieved with a fixed‐loop suspensory cortical button (EndoButton; Smith & Nephew), and tibial fixation was achieved with a cortical post technique using a double spike plate (DSP; Smith & Nephew), rather than an interference screw. Seven days after the operation, a radiograph of the knee demonstrated that the EndoButton had migrated into the femoral tunnel. The patient underwent prompt reoperation, and the EndoButton was manually repositioned and augmented by placing a large button onto it to prevent remigration.

**Conclusions:**

This case suggests that early postoperative intratunnel migration of a suspensory cortical button after PCL reconstruction can be successfully managed with prompt surgical reduction and augmentation.

## 1. Introduction

In the last decade, the treatment trend for acute posterior cruciate ligament (PCL) rupture has shifted from conservative to surgical [1]. Because 17%–66% of patients with PCL ruptures who are treated conservatively show progression of knee osteoarthritis [2], surgical techniques and methods for detailed anatomical evaluations of the PCL have been developed [3]. Suspensory cortical button fixation is widely used for femoral‐sided graft fixation in PCL reconstruction because of its procedural simplicity and reproducibility [3, 4]. Femoral cortical button malposition after PCL reconstruction is not uncommon; a postoperative CT study reported malposition (defined as a > 2‐mm gap between the button and cortex) in 17% of cases and identified male sex, low‐volume surgeons, concomitant ligament reconstruction, and fixed‐loop devices as risk factors [4]. Malposition refers to inadequate cortical seating (e.g., soft‐tissue interposition or a residual gap), whereas intratunnel migration represents postoperative displacement after initial fixation. Early postoperative intratunnel migration of a cortical button appears to be rare; here, an early migration of a suspensory cortical button (EndoButton) into the femoral tunnel after PCL reconstruction is described, along with a salvage technique.

## 2. Case Presentation

A 51‐year‐old man sustained a right knee injury while playing softball. Magnetic resonance imaging demonstrated an isolated PCL rupture without associated meniscal or chondral injury. On examination, the posterior sag sign was positive, and the posterior drawer was Grade II. Varus–valgus stress testing showed no laxity; the Lachman and anterior drawer tests were negative. Dial testing at 30° and 90° was symmetric, and the Slocum test was negative, indicating the absence of posterolateral corner or combined ligament injury. The patient completed at least 3 months of structured bracing and quadriceps‐strengthening rehabilitation but continued to experience persistent symptomatic posterior instability, particularly during stair descent. Given the ongoing symptoms despite nonoperative care and the patient′s activity goals, PCL reconstruction was performed.

### 2.1. First Operation

**Figure 1:**
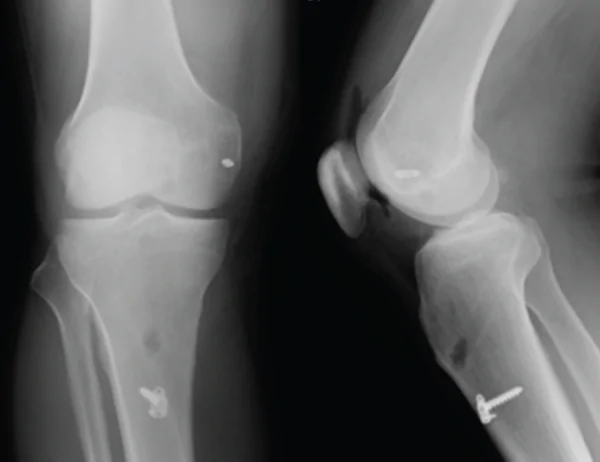
Anteroposterior and lateral radiographs of the right knee obtained immediately after surgery, showing the EndoButton positioned distal to the medial femoral epicondyle.

Under general anesthesia, an anatomic single‐bundle PCL reconstruction was performed using a quadrupled autologous semitendinosus graft. The patient was positioned supine, and the semitendinosus tendon was harvested through a small anteromedial incision. The quadrupled semitendinosus graft measured 7.5 mm at the femoral end and 8.0 mm at the tibial end, and no tapering was performed. Arthroscopically, the anterior cruciate ligament (ACL) appeared buckled within the intercondylar notch due to posterior tibial sag from PCL insufficiency rather than an ACL tear; probing confirmed intact fiber continuity and satisfactory tension. The remnant PCL was debrided using a radiofrequency device through the standard anteromedial and anterolateral portals and an additional posteromedial portal. Both 30° and 70° arthroscopes were used to visualize and prepare the tibial PCL footprint. Femoral and tibial tunnels were created via an outside‐in technique. Using the medial intercondylar ridge as the primary landmark and, when identifiable, the medial bifurcate ridge, the femoral socket center was positioned slightly anterolateral within the PCL footprint to reproduce the anterolateral fiber orientation commonly targeted in single‐bundle PCL reconstruction. Using an anteromedial entry femoral aimer (Smith & Nephew Endoscopy, Andover, Massachusetts, United States), a 2.4‐mm guidewire was placed at the planned socket center. A 4.0‐mm through‐hole was then created, followed by retrograde reaming of a 7.5‐mm‐diameter, 20‐mm‐deep femoral socket while preserving a 4.0‐mm cortical aperture for passage of the femoral suspensory cortical button. The tibial tunnel was created from an anteromedial tibial starting point using a tibial guide set at 60°. The guidewire tunnel was directed toward the center of the PCL facet on the posterior tibial plateau (the “champagne‐glass drop‐off” region), and its position and trajectory were confirmed fluoroscopically on both anteroposterior and lateral views. Then, the tunnel was reamed to 9.0 mm, 1 mm larger than the tibial end of the graft, to facilitate graft passage around the killer turn. The graft was passed through the tunnels and fixed on the femoral side using a fixed‐loop suspensory cortical button (EndoButton; Smith & Nephew, Andover, Massachusetts, United States). After cyclic loading, the knee was maintained at 90° of flexion, and one surgeon applied maximal manual anterior translation of the tibia. Although this reduction maneuver was maintained, the graft was tensioned to 40 N using a graft tensioner (Smith & Nephew), and the tibial side was then fixed with a double spike plate (DSP; Smith & Nephew, Andover, Massachusetts, United States) without an interference screw. Femoral cortical button seating and the final fixation construct were confirmed fluoroscopically (Figure 1). After completion of graft fixation and restoration of the posterior tibial position, arthroscopic assessment showed that the ACL appeared less buckled and more taut.

**Figure 2:**
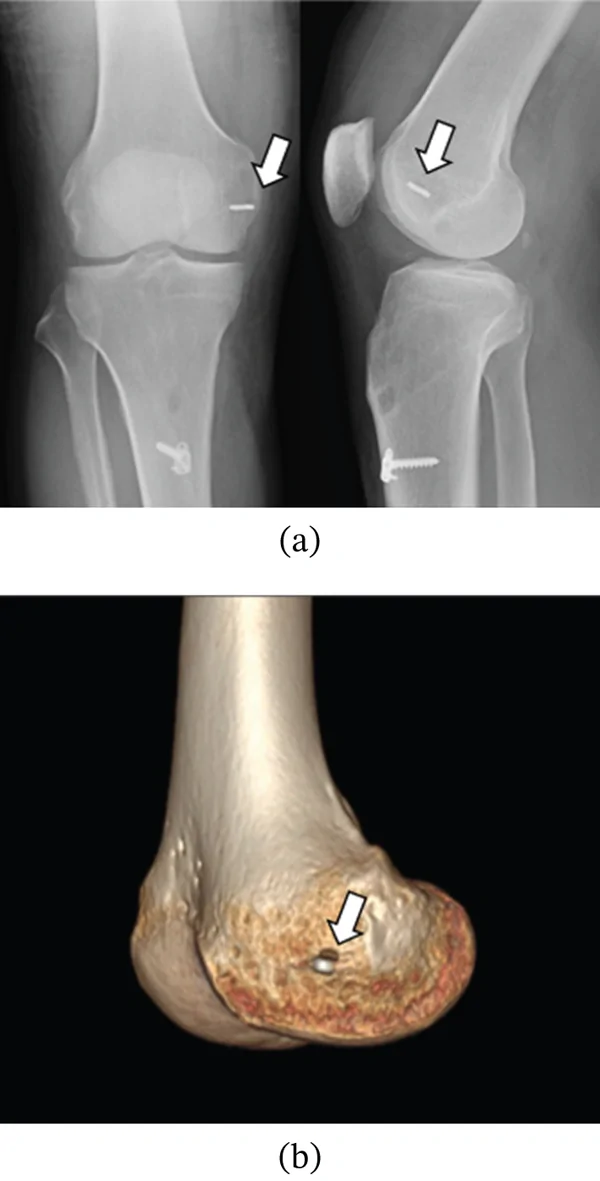
(a) Radiograph of the right knee seven (7) days after surgery showing that the EndoButton turned in a different direction and moved into the femoral tunnel (white arrow). (b) Three‐dimensional computed tomographic image demonstrating migration of the EndoButton into the femoral tunnel, with horizontal displacement of the button and a superior cortical defect/enlargement around the femoral tunnel outlet.

One week after the operation, knee flexion had progressed to 90°, and the patient was able to ambulate with full weight bearing. However, a routine postoperative radiograph obtained 1 week after surgery showed a change in the position and orientation of the EndoButton (Figure 2), and subsequent computed tomography demonstrated migration of the EndoButton into the femoral tunnel with enlargement of the femoral tunnel outlet (Figure 2). Accordingly, prompt revision surgery was performed.

### 2.2. Reoperation

**Figure 3:**
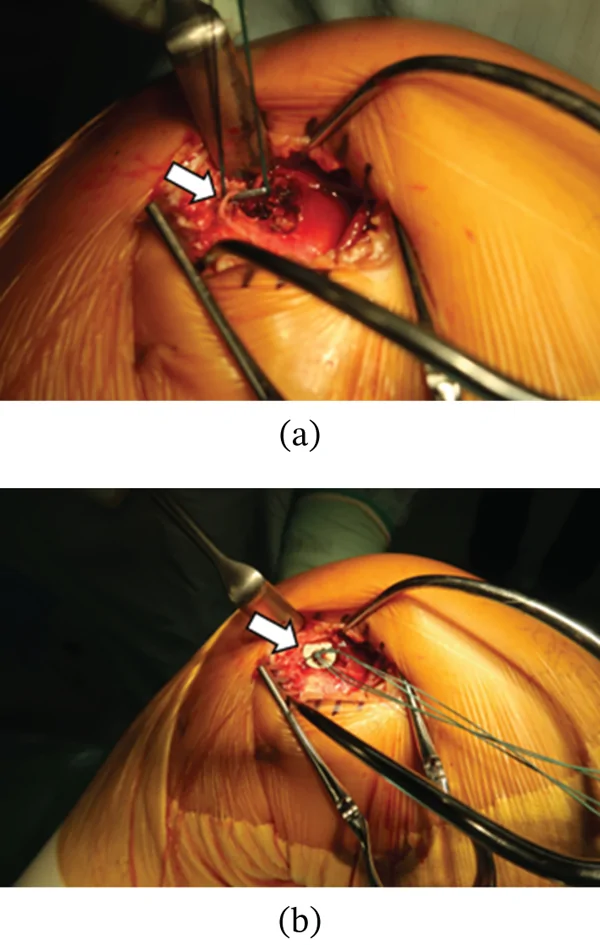
(a) Intraoperative photograph showing retrieval of the migrated EndoButton (white arrow) using a suture passed through the accessible button hole. (b) Photograph showing an augmentation by a TightRope ABS button (white arrow) on the repositioned EndoButton to prevent remigration.

**Figure 4:**
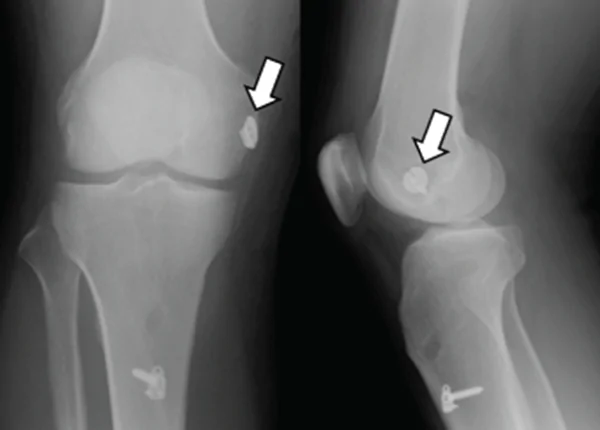
A radiograph of the right knee after revision surgery demonstrating that the repositioned EndoButton was augmented by TightRope ABS button (white arrow).

Under general anesthesia, an ~8‐cm medial knee incision was made. The vastus medialis was split, and the medial femoral condyle and femoral tunnel were exposed, allowing easy identification of the migrated EndoButton. The femoral tunnel outlet had enlarged owing to cortical breakage of the medial femoral condyle associated with button migration (Figure 3). Initially, only the superficial suture hole of the migrated EndoButton was accessible through the cortical defect. A No. Two braided polyester suture (e.g., #2 Ethibond Excel, Ethicon, Somerville, New Jersey, United States) was passed through this hole using mosquito forceps. With the knee flexed to 90°, traction was applied to the suture, allowing the EndoButton to be elevated toward the cortical surface. Once the second hole became accessible, a second no. 2 braided polyester suture was passed through it. Although traction was maintained on both sutures, the EndoButton was manipulated and flipped back onto the intact medial femoral cortex. After the reduction, a TightRope Attachable Button System (ABS) button (Arthrex, Naples, Florida, United States) was stacked onto the EndoButton by securing the #2 Ethibond sutures through the ABS slots. The 14‐mm ABS button provided sufficient cortical coverage to prevent remigration into the enlarged femoral tunnel outlet. The augmented construct remained stable and did not remigrate during full knee flexion and extension (Figure 4). Immediately after augmentation and final fixation, examination under anesthesia showed a negative posterior drawer with a firm endpoint and no posterior sag. Arthroscopy and revision of the tibial fixation, including DSP removal, had been planned if open reduction failed or adequate graft tension could not be restored. However, because open reduction and augmentation restored stable fixation and examination under anesthesia demonstrated a negative posterior drawer with a firm endpoint and no posterior sag, arthroscopy was not required.

After revision surgery, full weight bearing was permitted as tolerated. Range of motion was restricted to 0°–60° during the first postoperative week, 0°–90° from Weeks 1 to 3, and 0°–120° from Weeks 3 to 5. A PCL brace was worn except during rehabilitation sessions, without additional range‐of‐motion restriction through the brace. Quadriceps exercises were initiated immediately after revision surgery, and closed kinetic chain exercises, including squats, were started 3 weeks postoperatively.

At the 1‐year follow‐up, the DSP was removed because of local skin irritation caused by its subcutaneous prominence. Second‐look arthroscopy performed at the time of DSP removal demonstrated an intact PCL graft with satisfactory tension. Clinically, the posterior drawer test was negative, the posterior sag had resolved, and there was no evidence of hardware migration.

## 3. Discussion

This report describes a rare case of early postoperative intratunnel migration of a suspensory cortical button (EndoButton) after PCL reconstruction. Prompt open reduction of the migrated button and augmentation with a larger cortical button (TightRope ABS) provided an effective salvage procedure. Although suspensory cortical button fixation is widely used, femoral tunnel position and tunnel‐exit morphology should be carefully considered to minimize the risk of cortical failure and button migration.

Over the past decade, the number of PCL reconstructions has increased to improve functional instability and to reduce the risk of progressive osteoarthritis [5]. Shino et al. [3] reported arthroscopic PCL reconstruction surgery using suspensory cortical button devices including EndoButton. Suspensory cortical button fixation is widely used for femoral graft fixation in cruciate ligament reconstruction, although its biomechanical performance varies according to device design and testing conditions [3, 6]. However, complications such as postoperative suspensory cortical button migration into the knee joint [7–9] and mispositioning [10, 11] have been reported in ACL reconstruction surgery. Akaoka et al. [8] reported a case of early postoperative EndoButton migration into a femoral tunnel after ACL reconstruction, which was treated by early surgical reduction of the EndoButton and knot augmentation to prevent remigration. In the present case, early EndoButton migration occurred after PCL reconstruction. Two factors were key to successful salvage. First, early revision before complete intratunnel migration enabled direct identification and retrieval of the migrated EndoButton, as reported in an ACL reconstruction case [8]. The button was retrieved under direct visualization using two no. 2 braided polyester sutures passed sequentially through the accessible button holes. Second, augmentation was required to prevent remigration. Although a low‐profile button extender was considered, there was insufficient working space to pass an extender beneath the EndoButton. Therefore, the EndoButton was reduced onto an intact cortical segment and augmented by stacking a 14‐mm TightRope ABS button on top, securing the #2 Ethibond sutures through the ABS slots to increase the cortical footprint and prevent remigration. The 14‐mm TightRope ABS button provided sufficient cortical coverage of the enlarged femoral tunnel outlet. This technique is simple and may help restore stable femoral fixation. However, it may not be feasible if the button cannot be retrieved; therefore, an alternative strategy (e.g., femoral fixation with an interference screw) should be prepared for revision surgery. Furthermore, because the TightRope ABS construct is relatively bulky, this technique may be more suitable for PCL reconstruction—where the femoral exit lies beneath the vastus medialis—than for ACL reconstruction, in which the button is typically beneath the iliotibial band and may cause friction. In summary, early intervention with TightRope ABS augmentation may be considered a salvage option for early postoperative intratunnel migration of a suspensory cortical button after PCL reconstruction. In the present case, cortical failure and enlargement of the femoral tunnel outlet were observed in association with intratunnel migration of the EndoButton. Uchida et al. [12] reported that an EndoButton located posterior to the lateral supracondylar line migrated more frequently after anatomic double‐bundle ACL reconstruction, possibly because secure seating is difficult on the complex bony surface around the lateral epicondyle and near the attachments of the popliteus tendon and gastrocnemius. Similarly, in PCL reconstruction, suspensory cortical button fixation may be affected by the morphology of the femoral tunnel exit and the surrounding cortex.

In the present case, the femoral socket was created using a retrograde reaming technique, with preservation of a nominal 4.0‐mm cortical aperture. Therefore, the initial 4.0‐mm cortical aperture was not considered excessively large relative to the 12‐mm EndoButton used at the index procedure. However, the oblique relationship between the tunnel and the medial femoral cortical surface may have resulted in uneven contact between the button and the surrounding cortex, concentrating load on a limited portion of the cortical rim. Repetitive loading during the early postoperative period may subsequently have caused cortical failure and enlargement of the defect, ultimately allowing further intratunnel migration of the EndoButton.

Based on biomechanical considerations reported for ACL reconstruction, a similar principle may apply to PCL reconstruction. To reduce the risk of cortical failure, the femoral tunnel exit should preferably be located proximal to the medial epicondyle on a broad cortical surface and oriented as perpendicular as reasonably possible to the medial femoral cortex while maintaining anatomic placement of the femoral socket. Although the exact mechanism of cortical failure could not be determined, care should be taken to minimize unnecessary trauma to the cortical aperture during outside‐in femoral tunnel preparation. Excessive pressure exerted by the guide or sleeve against the medial femoral cortex should be avoided. Finally, intraoperative fluoroscopy or direct visualization may help confirm appropriate deployment and cortical seating of the femoral button [13].

## 4. Conclusions

This case suggests that early postoperative intratunnel migration of a suspensory cortical button after PCL reconstruction can be successfully managed with prompt surgical reduction and augmentation.

## Author Contributions

Daichi Ishimaru was the primary surgeon and contributed to manuscript preparation. Kazuki Sohmiya performed the surgery. Kazu Matsumoto analyzed the situation of the knee and supervised the drafting. Nobuo Terabayashi supervised the drafting and revision of the manuscript and provided critical feedback.

## Funding

No funding was received for this manuscript.

## Disclosure

All authors read the manuscript and approved it.

## Consent

The patient was informed and gave consent for the case report to be published. The ethics board at the authors′ institution approved the presentation of this case report.

## Conflicts of Interest

The authors declare no conflicts of interest.

## Data Availability

The data that support the findings of this study are available on request from the corresponding author. The data are not publicly available due to privacy or ethical restrictions.
